# A phase II, non-comparative randomised trial of two treatments involving liposomal amphotericin B and miltefosine for post-kala-azar dermal leishmaniasis in India and Bangladesh

**DOI:** 10.1371/journal.pntd.0012242

**Published:** 2024-06-20

**Authors:** Shyam Sundar, Krishna Pandey, Dinesh Mondal, Major Madhukar, Roshan Kamal Topno, Ashish Kumar, Vinod Kumar, Deepak Kumar Verma, Jaya Chakravarty, Rahul Chaubey, Poonam Kumari, Md. Utba Rashid, Shomik Maruf, Prakash Ghosh, Sheeraz Raja, Joelle Rode, Margriet den Boer, Pradeep Das, Jorge Alvar, Suman Rijal, Fabiana Alves

**Affiliations:** 1 Kala-azar Medical Research Center (KARMC), Muzaffarpur, India; 2 Institute of Medical Sciences, Banaras Hindu University, Varanasi, India; 3 ICMR-Rajendra Memorial Research Institute of Medical Sciences (RMRIMS), Patna, India; 4 International Centre for Diarrhoeal Disease Research, (icddrb,b), Dhaka, Bangladesh; 5 Drugs for Neglected Diseases initiative, New Delhi, India; 6 Drugs for Neglected Diseases initiative, Rio de Janeiro, Brazil; 7 Médecins Sans Frontières (MSF), Amsterdam, The Netherlands; 8 Drugs for Neglected Diseases initiative, Geneva, Switzerland; Institute of Postgraduate Medical Education and Research, INDIA

## Abstract

**Background:**

In Southeast Asia, treatment is recommended for all patients with post-kala-azar dermal leishmaniasis (PKDL). Adherence to the first-line regimen, twelve weeks of miltefosine (MF), is low and ocular toxicity has been observed with this exposure period. We assessed the safety and efficacy of two shorter-course treatments: liposomal amphotericin B (LAmB) alone and combined with MF.

**Methodology/Principal findings:**

An open-label, phase II, randomized, parallel-arm, non-comparative trial was conducted in patients with parasitologically confirmed PKDL, 6 to ≤60 years. Patients were assigned to 20 mg/kg LAmB (total dose, in five injections over 15 days) alone or combined with allometric MF (3 weeks). The primary endpoint was definitive cure at 12 months, defined as complete resolution of papular and nodular lesions and >80% re-pigmentation of macular lesions. Definitive cure at 24 months was a secondary efficacy endpoint. 118/126 patients completed the trial. Definitive cure at 12 months was observed in 29% (18/63) patients receiving LAmB and 30% (19/63) receiving LAmB/MF (mITT), increasing to 58% and 66%, respectively, at 24 months. Most lesions had resolved/improved at 12 and 24 months for patients receiving LAmB (90%, 83%) and LAmB/MF (85%, 88%) by qualitative assessment. One death, unrelated to study drugs, was reported; no study drug-related serious adverse events were observed. The most frequent adverse drug reactions were MF-related vomiting and nausea, and LAmB-related hypokalaemia and infusion reactions. Most adverse events were mild; no ocular adverse events occurred.

**Conclusions/Significance:**

Both regimens are suitably safe and efficacious alternatives to long-course MF for PKDL in South Asia.

**Trial registration:**

CTRI/2017/04/008421.

## Author summary

Post-kala-azar dermal leishmaniasis (PKDL) is a skin rash which can appear after successful treatment of visceral leishmaniasis caused by the parasite *Leishmania donovani*. Although not life-threatening for the patient, this condition can be associated with social stigma, and the sandfly that transmits the disease can become infected after feeding on patients with PKDL lesions. Therefore, all patients are treated to control visceral leishmaniasis within the Southeast Asia kala-azar elimination initiative. It is difficult to justify the use of existing treatments in patients who are otherwise generally well, since they are prolonged, potentially toxic, and expensive. We designed a trial at two sites in India and one in Bangladesh to assess the efficacy and safety of two treatment options for patients with PKDL: 20 mg/kg liposomal amphotericin B in monotherapy and 20 mg/kg liposomal amphotericin B in combination with three weeks of miltefosine.

These two regimens were found to have a similar efficacy to the current first line treatment, but with a much shorter treatment duration, which also reduces the risk of side-effects and makes them particularly useful in the current context of elimination in the region.

## Introduction

Post-kala-azar dermal leishmaniasis (PKDL) is a skin rash presenting in 5–15% of patients in the Indian subcontinent (ISC), usually months to years after treatment of visceral leishmaniasis (VL) [1,2]. It mostly manifests as mild macular and/or papular skin lesions, and is of little consequence to patients, although severe nodular presentations may occur and untreated lesions usually persist for years. The sandfly vector, *P*. *argentipes*, can become infected after feeding on people with PKDL [3,4]. Given this potentially important role in transmission, people with PKDL should be actively identified and offered treatment within the Southeast Asia kala-azar elimination initiative. We urgently need a safe, short, and cost-effective treatment regimen for PKDL in South Asia.

Historically, it has been difficult to convince otherwise well people with PKDL in the ISC to accept previously available treatments, sodium stibogluconate and amphotericin B deoxycholate, which are prolonged, painful, and expensive [5,6]. A limitation on PKDL trials searching for new therapies is the lack of a standardized objective method to define PKDL cure; therefore, the results of different studies need to be interpreted and compared with caution. Following a small trial (n = 18 per arm) that observed a 93% cure rate in patients presenting papular or nodular PKDL, the WHO recommended 12 weeks of miltefosine (MF) for PKDL in the ISC as a first option treatment [7]. Adherence to this regimen has been hampered by the long duration and gastro-intestinal side effects [8,9], and current data suggest that MF-related ocular toxicity occurs in up to 3.7% of PKDL patients, including cases of permanent partial vision loss [10]. Other issues include potential teratogenicity, making contraception a requirement for six months and mandatory pregnancy testing before administration [8,9].

A second treatment option, liposomal amphotericin B (LAmB) monotherapy, has been successfully used to treat PKDL in India and Bangladesh. In closely monitored patient sets, a 30 mg/kg total dose cured most patients at 12 months in India (82%, 59/72) and Bangladesh (80%, 70/88) [11,12]. However, 30 mg/kg LAmB was associated with severe hypokalaemia, and with rhabdomyolysis in a small case series in Bangladesh [13].

In the current study, two treatment regimens were evaluated: 1) LAmB 20 mg/kg total dose as monotherapy and 2) LAmB 20 mg/kg total dose in combination with three weeks of MF. LAmB was administered in five divided doses over 15 days because parasite clearance is faster when LAmB is administered in multiple injections compared to a single administration [14]. The 20 mg/kg LAmB regimen was chosen to maximize the anti-parasitic effect, while minimizing the safety risks of severe hypokalaemia and rhabdomyolysis. As MF has a seven-day half-life and reaches steady state after four weeks, a three-week treatment was considered necessary for sufficient exposure.

The primary objective was to assess the safety and efficacy of both regimens at 12 months. A secondary objective was to evaluate patients at 24 months, given the time required for skin re-pigmentation and the occurrence of late relapses (> 12 months post-therapy) in previous PKDL trials with LAmB and MF [8,15,16].

## Methods

### Ethics statement

Approvals were obtained from institutional ethics committees (IECs) at the Kala-azar Medical Research Center (KAMRC), Muzaffarpur, Rajendra Medical Research Institute of Medical Sciences (RMRIMS), Patna, and the Surya Kanta Kala-azar Research Centre, International Centre for Diarrhoeal Disease Research, Bangladesh (icddr,b). Two protocol amendments were developed, endorsed by the Data Safety Monitoring Board (DSMB) and approved by the IECs: one to adjust the sample size of the study by 15% (from 110 to 126) due to higher than expected frequency of allergic reactions related to LAmB after enrolment of the initial patients, requiring permanent treatment discontinuation and rescue therapy, and replacement of the LAmB batch, and a second amendment extending follow-up to 24 months. The study was conducted in accordance with the Declaration of Helsinki, International Council for Harmonization Good Clinical Practice guidelines, and all applicable state, local, and foreign laws for protecting the rights and welfare of human subjects. Informed consent and assent (when applicable) were obtained as per regulatory requirements. Written voluntary informed consent was obtained from adult patients and from the parent or guardian of any child <18 years old; assent from minors was also obtained according to country regulations.

This trial is registered at the Indian registry of clinical trials (CTRI/2017/04/008421). The protocol is available online [17].

### Study design and participants

This open-label, phase II, randomized, parallel-arm, non-comparative trial was conducted at two sites in India: The Kala-azar Medical Research Center (KAMRC), Muzaffarpur and Rajendra Medical Research Institute of Medical Sciences (RMRIMS), Patna; and one site in Bangladesh: Surya Kanta Kala-azar Research Centre, International Centre for Diarrhoeal Disease Research (icddr,b). It was designed as a non-comparative trial, due to the unfeasibility of implementing a 3-arm comparative study with the standard of care in the current context of VL elimination in South Asia. Patients were randomized 1:1 to either LAmB monotherapy or LAmB in combination with MF. Patients aged 6 to ≤60 years with clinical presentation of PKDL and confirmatory diagnosis through demonstration of parasites in a skin smear by microscopy or PCR were included. Exclusion criteria included PKDL treatment within the last two years, being pregnant or lactating, women of childbearing potential who did not accept to use contraception from the beginning of the treatment until five months after the end of treatment (EOT) due to long half-life of miltefosine, signs and symptoms of other severe diseases, severe malnutrition, haemoglobin <5 g/dL, abnormal liver function (alanine aminotransferase and aspartate aminotransferase tests >3 times upper limit of normal (ULN)), total bilirubin levels >1.5 times ULN, serum creatinine above ULN, serum potassium <3.5 mmol/L, history of allergy or hypersensitivity to the study drugs, a positive HIV test, and being on immunomodulator therapy.

### Procedures

Arm A comprised LAmB (AmBisome, Gilead Sciences, USA) monotherapy, 5 x 4 mg/kg on days 1, 4, 8, 11, and 15 (total dose of 20 mg/kg). Arm B comprised LAmB (as per arm A) in combination with MF (Impavido, Knight Therapeutics, Canada) orally BID for 21 days, starting concomitantly from day 1. Children ≤30 kg received allometric MF dosing based on sex, weight, and height [18]; patients >30 kg to <45kg received 100 mg/day, and patients ≥45 kg received 150 mg/day; doses were administered BID with food.

Patients were hospitalized for 15 days during LAmB treatment; after discharge, MF treatment was continued on an out-patient basis for 1 additional week for patients in arm B with daily telephone contact and patients instructed to return empty blisters to the clinic. Full adherence was assumed if 90–110% of prescribed doses were administered.

Rescue treatment, consisting of 12 weeks of MF monotherapy, was indicated in case of: failure to respond to treatment, reappearance/reactivation of lesions after an initial improvement, lesions worsening over time, or intolerance to LAmB.

### Assessments

Safety assessments, including clinical history, physical examination and vital signs, haematological and biochemical laboratory investigations were carried out in the screening period (Day -28 to day 0) and on Days 1, 8, 15, and 30; and additionally on Day 22 for patients in arm B only (after last day of MF). At the time of the study implementation, the WHO recommendations to minimize the risk of ocular events following MF administration had not yet been released [19]. Ocular investigations were based on clinical history and physical examination, as for other body systems. All AEs were classified by the Investigator as related or not related to the study drugs, based on the well-known safety profiles of LAmB and miltefosine, and the timing of the events.

Initial clinical assessment for efficacy was made at day 30 (EOT). All patients had follow-up visits at 3, 6, 12, and 24 months after treatment initiation to assess safety and efficacy.

Efficacy was assessed by two methods. In the first, clinical evolution was systematically recorded semi-quantitatively [20]. PKDL lesions were characterized clinically during the screening period (Days -28 to 0), at day 30, and at the 3, 6, 12, and 24-month follow-up visits. Using the scoring system described by Mondal et al, skin lesions were plotted in squares corresponding to the affected areas in the body map [20]. The total number of squares in the body map containing PKDL lesions (PKDL score) was counted at screening, day 30, and at 3, 6, 12, and 24-month follow-ups, and the proportion of improvement in relation to baseline was calculated based on:

Percentage of skin lesions cured = number of squares affected at screening—number of squares affected after treatment x 100/ total number of squares affected at screening.

A second method was a qualitative assessment of lesion improvement by the Investigators. Lesions were photographed and qualitatively assessed by investigators’ visual evaluation at 12 and at 24 months, according to four categories adapted from den Boer et al. [11]. A blinded reviewer revised the photographs, and discrepancies were assessed and resolved.

- Category 1: All lesions have improved and the majority of lesions have resolved. Any remaining lesions are only faintly visible as re-pigmentation is almost complete.
- Category 2: The majority of lesions show significant improvement, some lesions have resolved.
- Category 3: No new lesions, but the majority of lesions show no or slight improvement.
- Category 4: New lesions have appeared and there is no or little improvement of other lesions

### Endpoints

The primary efficacy endpoint was definitive cure at 12 months after treatment onset, defined as complete resolution of papular and nodular lesions (flattening of 100% of lesions) and significant improvement (>80% re-pigmentation) of macular lesions; patients reaching definitive cure were considered responders. The percentage improvement was determined based on the semi-quantitative PKDL scoring system described above [20]. Patients not meeting criteria for definitive cure (non-responders) were categorised according to the extent of lesion improvement (≤70%, >70%). Due to the lengthy process of complete skin re-pigmentation, an additional post-hoc analysis considered >70% re-pigmentation of macular lesions as clinically meaningful for assessment of PKDL treatment response, this was defined in the SAP prior to data analysis. Secondary efficacy endpoints included definitive cure (as defined above) at 24 months and the investigators’ qualitative assessment to characterize the clinical evolution of PKDL lesions at 12 and 24 months.

Safety endpoints were serious adverse events (SAEs) from start of treatment to 24-month follow-up; frequency and severity of adverse events (AEs) that led to treatment discontinuation, and frequency and severity of AEs from start of treatment to six-month follow-up. Between six and 24 months’ follow-up, safety reporting was limited to all SAEs and AEs related to study drugs or study procedures.

### Statistical analysis

A minimum of 50 patients per treatment arm was estimated to provide a precision estimate of 10% with 95% CI, based on an anticipated cure rate of 85% at 12 months. Thirteen patients were added to allow for 10% loss during follow-up and 15% discontinuing treatment (due to an unexpectedly high occurrence of LAmB allergic reactions in the first group of patients recruited), resulting in a sample size of 63 patients per treatment arm and an overall sample size of 126. This study was non-comparative.

A computer-generated randomization code was used for patient treatment allocation to one of the two treatment arms in a 1:1 ratio. A set of individual opaque, sealed, and sequentially numbered envelopes was provided to the treatment sites, with one envelope per patient containing the treatment allocation.

Primary and secondary efficacy analyses were performed on the modified intention-to-treat (mITT, all randomized patients receiving at least one dose of treatment; in case of error in treatment allocation, the actual treatment received was used in the analysis) and per protocol (PP, patients in the mITT with no major protocol deviations) population sets. Primary and secondary efficacy evaluations were performed on both mITT and PP populations with a worst-case scenario applied (missing efficacy outcome at 12 and/or 24 months was considered as treatment failure) and with the set of completers (patients who did not have an outcome at 12 and/or 24 months were excluded from the analysis). Efficacy endpoints were reported as the proportion of cured patients with a corresponding 95% confidence interval (CI).

The safety analysis was performed on the mITT set, based on treatment-emergent adverse events (TEAEs). The number of patients presenting TEAEs was summarized by treatment arm. AEs were categorized by system organ class and preferred term according to the Medical Dictionary for Regulatory Activities Version 24.0, severity (based on the Common Terminology Criteria for Adverse Events, v4.03), seriousness, and causal relationship to study drugs.

### Role of the funding source

The funders of the study (see acknowledgements) had no role in study design, data collection, data analysis, data interpretation, or writing of the report.

## Results

### Study population

**Fig 1:**
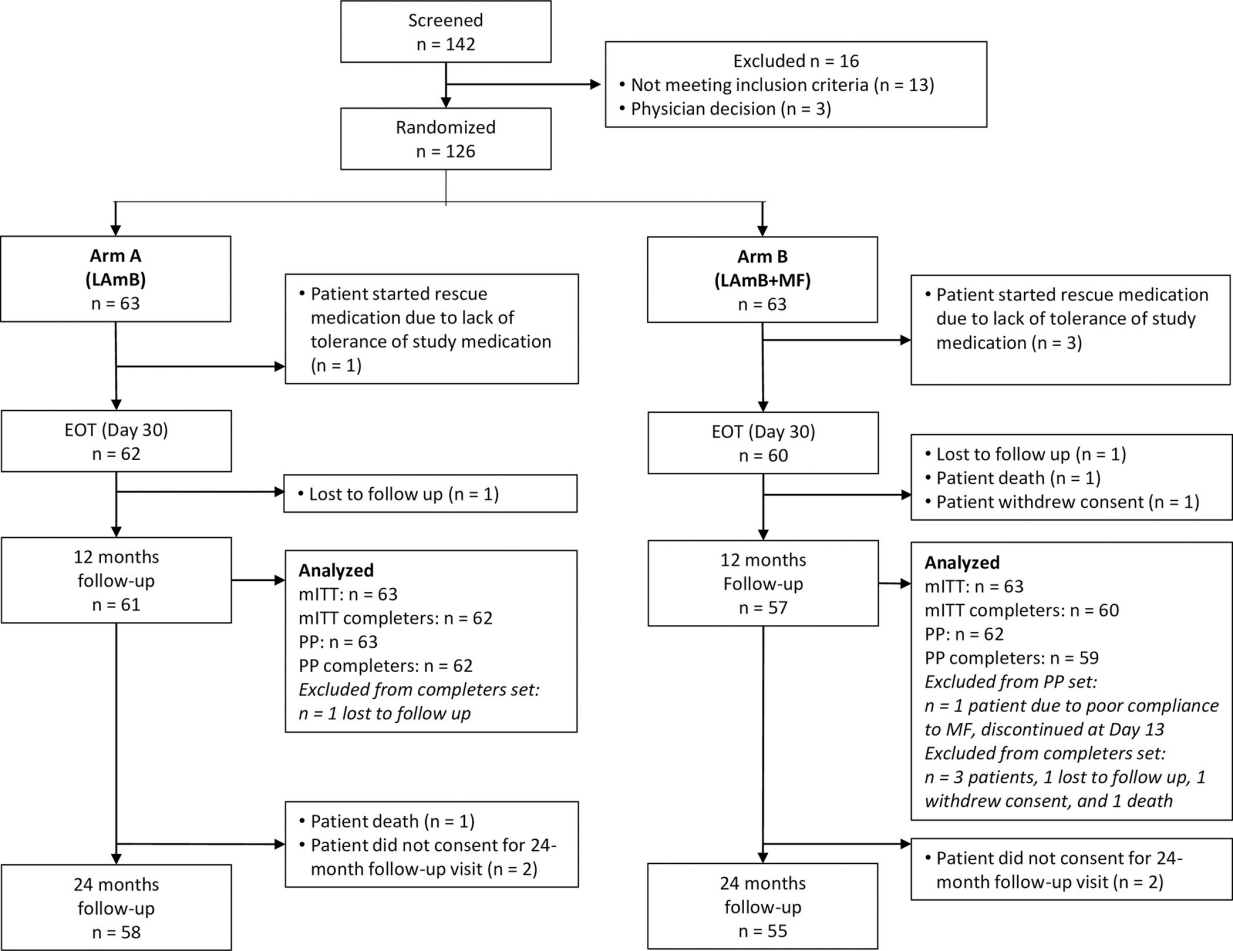
Participant flow chart. FUL = follow up, M = month.

The study was conducted between 22 November 2017 and 13 April 2021. A total of 142 patients were screened and 63 were assigned to each treatment arm, making a total of 126 patients enrolled in the study: 50 patients were recruited in KAMRC site, 46 in RMRIMS site and 30 patients in iccdr,b site. Overall, 121/126 patients reached EOT and 118/126 (94%) reached the 12-month follow-up visit (Fig 1). Four patients needed rescue treatment because of intolerance to study treatment (one in arm A, three in arm B). Two patients were lost to follow-up (one in each arm), one patient withdrew consent (arm B) and one patient died (arm B). 113/126 patients attended the 24-month follow-up visit. The two patients lost to follow-up at 12 months, and four others, did not consent to a 24-month follow-up visit, and one patient died after completing the 12-month follow-up visit (Fig 1). For the primary 12-month efficacy evaluation, all patients were included in the mITT set and 125/126 of patients in the PP set (MF was not administered after day 13 in one patient in arm B, this patient did not require rescue therapy and continued in the study). Four patients did not have an outcome at 12 months (arm A: one lost to follow-up; arm B: one lost to follow-up, one death and one consent withdrawal), thus 122/126 (97%) and 121/126 (96%) patients were included in the completers set for the mITT and PP sets, respectively (Fig 1). 113/126 patients completed the 24-month follow-up visit and 118/126 patients were included in the mITT. Patients who received rescue treatment due to AE or treatment failure at 12 months were included in the analysis and considered as failures.

Demographic and baseline characteristics were similar between treatment arms, except for age; the median age was 25 years, with 29 and 22 years in arms A and B, respectively (p = 0.0378). The higher proportion of male patients (56%) is consistent with the reported cases of VL in the region.

Most patients had a previous history of diagnosis and treatment of VL (106/126, 84%) and 12/13 patients who had received previous treatment for PKDL, ≥2 years prior to enrolment, were from the icddr,b site. 9/126 (7%) patients presented with papular and nodular lesions, 54/126 (43%) with macular, and 63/126 (50%) with mixed-pattern lesions (macular and papular/nodular). The lesion types were equally distributed and PKDL scores were similar between treatment arms (Table 1). Baseline laboratory parameters were also similar between arms and overall within normal values range (Table A in S1 Text).

**Table 1:** Demographic data and baseline characteristics.

	Statistics	Arm A (LAmB) (N = 63)	Arm B (LAmB/MF) (N = 63)	Total (N = 126)
Age (years)	N	63	63	126
	Mean (SD)	31 (15.2)	26 (12.8)	28 (14.2)
	Median	29	22	25
	Min, Max	7, 58	10, 59	7, 59
Sex*				
Male	N (%)	37 (58.73%)	34 (53.97%)	71 (56.35%)
Female	N (%)	26 (41.27%)	29 (46.03%)	55 (43.65%)
Height (cm)	N	63	63	126
	Mean (SD)	155.3 (13.81)	154.3 (10.31)	154.8 (12.15)
	Median	155.0	155.0	155.0
	Min, Max	107.4, 180.0	122.0, 180.0	107.4, 180.0
Weight (kg)	N	63	63	126
	Mean (SD)	49.2 (12.35)	46.8 (10.07)	48.0 (11.29)
	Median	50.0	45.0	47.8
	Min, Max	15.0, 80.0	25.0, 75.0	15.0, 80.0
Nutritional Status according to WHO reference curves and Body Mass Index**	Underweight N (%)	16 (25.40%)	23 (36.51%)	39 (30.95%)
	Normal weight N(%)	41 (65.08%)	36 (57.14%)	77 (61.11%)
	Overweight N (%)	6 (9.52%)	4 (6.35%)	10 (7.94%)
Types of lesions				
Papular and nodular lesions	N (%)	4 (6.35%)	5 (7.94%)	9 (7.14%)
Macular lesions	N (%)	27 (42.86%)	27 (42.86%)	54 (42.86%)
Mixed lesions	N (%)	32 (50.79%)	31 (49.21%)	63 (50.00%)
PKDL score***	N	63	63	126
		Mean (SD)	144 (120.6)	172 (142.1)	158 (132.0)
		Median	150	165	154
		Min, Max	2, 487	3, 534	2, 534

*Based on self-report.

** Nutritional status defined by BMI for age WHO reference curves for gender for participants 6–19 years; and BMI for subjects > 19 years old.

*** PKDL score: total number of squares in the body map containing PKDL lesions

LAmB = liposomal amphotericin B, MF = miltefosine.

Only patients who discontinued treatment due to an AE did not receive the full treatment: four patients experienced hypersensitivity to LAmB (withdrawn and received rescue therapy) and one with MF-related vomiting in arm B (MF stopped after Day 13, did not receive rescue therapy and continued in the study).

### Efficacy

At 12 months, 29% and 30% of patients had definitive cure in arms A and B, respectively, in the mITT analysis. Similar results were observed in the mITT complete case and PP analyses. Responders and patients with an improvement of >70% compared to baseline comprised 46% (29/63) and 51% (32/63) of patients in arms A and B, respectively (Table 2). A sensitivity analysis in the set of completers (excluding any missing 12-month efficacy outcomes) yielded similar results with 29/62 (47%) of patients in arm A and 32/60 (53%) in arm B in the mITT set (Table B in S1 Text). At 12 months, seven patients had received rescue therapy: four due to LAmB-related AE, requiring treatment discontinuation, and three due to treatment failure.

**Table 2:** Proportion of patients who met the definitive cure criteria at 12 months based on the semi-quantitative method, and % of improvement among non-responders–worst case analysis.

Outcome	Statistics	Arm A (LAmB) (N = 63)	Arm B (LAmB/MF) (N = 63)	Total (N = 126)
Responder*	n (%)	18 (28.57%)	19 (30.16%)	37 (29.37%)
	95% CI	(17.42%, 39.73%)	(18.83%, 41.49%)	(21.41%, 37.32%)
Non-responder	n (%)	45 (71.43%)	44 (69.84%)	89 (70.63%)
	95% CI	(60.27%, 82.58%)	(58.51%, 81.17%)	(62.68%, 78.59%)
% of improvement for non-responder				
≤70% improvement	n (%)	32 (50.79%)	25 (39.68%)	57 (45.23%)
	95% CI	(38.45%, 63.14%)	(27.60%, 51.76%)	(36.55%, 53.93%)
>70% improvement	n (%)	11 (17.46%)	13 (20.63%)	24 (19.05%)
	95% CI	(8.09%, 26.83%)	(10.64%, 30.63%)	(12.29%, 25.90%)

LAmB = liposomal amphotericin B, MF = miltefosine, N = number of patients, CI = confidence interval. Note: Patients with missing data at 12 months were not included in the categorization of non-responders. *Completely resolved all papular and nodular lesions and >80% re-pigmentation of macular lesions.

Patients classified as non-responders at 12 months were still progressing with lesion re-pigmentation, therefore, investigators did not initiate rescue therapy and continued assessment until the 24-month follow-up.

Lesion re-pigmentation continued to progress and, at 24 months, the proportion of patients with definitive cure increased to 58% and 66% for arms A and B, respectively. When considering responders and those with >70% improvement, more than 70% of patients responded to therapy (73% and 80% in arms A and B, respectively) (Table 3). Similar results were observed with mITT complete case and PP analyses. Overall, patients with only macular lesions at baseline had a better definitive cure rate at 12 and 24 months than patients with mixed lesions (Table C in S1 Text). More patients relapsed between 12 and 24 months in arm A than arm B (five and one, respectively).

**Table 3:** Proportion of patients who met the definitive cure criteria at 24 months based on the semi-quantitative method, and % of improvement among non-responders–worst case analysis.

Outcome	Statistics	Arm A (LAmB) (N = 59)	Arm B (LAmB/MF) (N = 59)	Total (N = 118)
Responder*	n (%)	34 (57.63%)	39 (66.10%)	73 (61.86%)
	95% CI	(45.02%, 70.24%)	(54.02%, 78.18%)	(53.10%, 70.63%)
Non-responder	n (%)	25 (42.37%)	20 (33.90%)	45 (38.14%)
	95% CI	(29.76%, 54.98%)	(21.82%, 45.98%)	(29.37%, 46.90%)
% of improvement for non-responder				
≤70% improvement	n (%)	13 (22.03%)	7 (11.86%)	20 (16.95%)
	95% CI	(11.46%, 32.61%)	(3.61%, 20.12%)	(10.18%, 23.72%)
>70% improvement	n (%)	9 (15.25%)	8 (13.56%)	17 (14.41%)
	95% CI	(6.08%, 24.43%)	(4.82%, 22.30%)	(8.07%, 20.74%)

LAmB = liposomal amphotericin B, MF = miltefosine, N = number of patients, CI = confidence interval. *Note*: Patients with missing data at 24 months were not included in the categorization of non-responders. *Completely resolved all papular and nodular lesions and >80% re-pigmentation of macular lesions.

Using the investigator’s qualitative assessment, most lesions resolved or improved significantly (categories 1+2) at 12 months in 90% (56/62) of patients in arm A and 85% (51/60) of patients in arm B. At 24 months, this changed to 83% (49/59) of patients in arm A and 88% (51/58) of patients in arm B (Table 4).

**Table 4:** Qualitative assessment of PKDL at 12 and 24 months–set of completers.

Visit	Category	Statistics	Arm A (LAmB) (N = 62) n (%)	Arm B (LAmB/MF) (N = 60) n (%)	Total (N = 122) n (%)
12 Months	1	n (%)	38 (61.29%)	40 (66.67%)	78 (63.93%)
		95% CI	(49.17%, 73.41%)	(54.74%, 78.59%)	(55.41%, 72.46%)
	2	n (%)	18 (29.03%)	11 (18.33%)	29 (23.77%)
		95% CI	(17.73%, 40.33%)	(8.54%, 28.12%)	(16.22%, 31.32%)
	3	n (%)	2 (3.23%)	3 (5.00%)	5 (4.10%)
		95% CI	(0.00%, 7.62%)	(0.00%, 10.51%)	(0.58%, 7.62%)
	4	n (%)	3 (4.84%)	3 (5.00%)	6 (4.92%)
		95% CI	(0.00%, 10.18%)	(0.00%, 10.51%)	(1.08%, 8.76%)
			**(N = 59)** **n (%)**	**(N = 58)** **n (%)**	**(N = 117)** **n (%)**
24 Months	1	n (%)	44 (74.58%)	45 (77.59%)	89 (76.07%)
		95% CI	(63.47%, 85.69%)	(66.85%, 88.32%)	(68.34%,83.80%)
	2	n (%)	5 (8.47%)	6 (10.34%)	11 (9.40%)
		95% CI	(1.37%, 15.58%)	(2.51%, 18.18%)	(4.11%, 14.69%)
	3	n (%)	0 (0.00%)	1 (1.72%)	1 (0.85%)
		95% CI	(0.00%, 0.00%)	(0.00%, 5.07%)	(0.00%, 2.52%)
	4	n (%)	9 (15.25%)	3 (5.17%)	12 (10.26%)
		95% CI	(6.08%, 24.43%)	(0.00%,10.87%)	(4.76%, 15.75%)

LAmB = liposomal amphotericin B, MF = miltefosine

Category 1 = All lesions have improved, and the majority of lesions have resolved. Any remaining lesions are only faint.

Category 2 = The majority of lesions show significant improvement, some lesions have resolved.

Category 3 = No new lesions, but the majority of lesions show no or slight improvement.

Category 4 = New lesions have appeared and there is no or little improvement of other lesions

**Note:** Patients withdrawn for AE were included in set of completers at 12 and 24 months but were not included in the categorization of qualitative assessment.

### Safety

At least one TEAE occurred in 67/126 (53%) of patients, mostly during the treatment period or within 3 months of follow-up, with 18/63 (28%) and 49/63 (78%) in arms A and B, respectively. Most TEAEs were mild or moderate (CTCAE grade 1 or 2). Six severe TEAEs (CTCAE grade ≥3) were reported in six patients (4.8%) in arm B. Four of these events were considered related to the study drugs: hypokalaemia (one), creatinine increase (two), a decreased platelet count (one); and two events were not considered related to the study drugs: toothache (one), and one death of unknown cause investigated through verbal autopsy, which occurred 31 days after last dose of LAmB and 25 days after last dose of miltefosine. At least one adverse drug reaction was reported in 60/126 (48%) patients, 13/63 (21%) in arm A and 47/63 (75%) in arm B, the most common being vomiting (31%) and nausea (5.6%), mostly MF-related. Less common were LAmB-related back pain (7.1%), pyrexia (6.3%), and hypokalaemia (4.0%). The higher frequency of ADRs in arm B was mostly due to vomiting, with some patients vomiting on several days. Most ADRs were mild or moderate, no ocular toxicity was observed in the trial. One patient in arm A and three patients in arm B discontinued treatment because of LAmB-related hypersensitivity (three) and rash with pruritus (one), three events occurred during or after infusion of LAmB testing dose, and one occurred during the administration of first LAmB dose; all received rescue medication. One patient discontinued treatment in arm B because of MF-related vomiting; rescue treatment was not indicated by the Investigator, and the patient continued in the study. No ocular adverse events were observed. One death in arm B, unrelated to the study medication, was reported; no other SAEs were reported (Tables 5 and D in S1 Text). Despite receiving two Depo-Provera injections (at baseline and three-month follow-up), one case of pregnancy was reported in a patient in arm B, who delivered a healthy male at term 13 months after treatment onset.

**Table 5:** Summary of treatment emergent adverse events related to study drug by System Organ Class and Preferred Term (mITT population).

	Total		
	Arm A (LAmB) (N = 63) n (%) e	Arm B (LAmB/MF) (N = 63) n (%) e	
	**LAmB**	**LAmB**	**MF**
**System Organ Class Preferred Term**			
Patients with At least one TEAE	13 (20.63%) 19	22 (34.92%) 27	38 (60.32%) 143
Gastrointestinal disorders	0 (0.00%) 0	1 (1.59%) 1	38 (60.32%) 139
Abdominal pain upper	0 (0.00%) 0	0 (0.00%) 0	1 (1.59%) 1
Diarrhoea	0 (0.00%) 0	0 (0.00%) 0	1 (1.59%) 1
Gastritis	0 (0.00%) 0	0 (0.00%) 0	1 (1.59%) 1
Nausea	0 (0.00%) 0	0 (0.00%) 0	6 (9.52%) 7
Vomiting	0 (0.00%) 0	1 (1.59%) 1	38 (60.32%) 129
General disorders and administration site conditions	6 (9.52%) 9	4 (6.35%) 4	0 (0.00%) 0
Chills	1 (1.59%) 1	1 (1.59%) 1	0 (0.00%) 0
Pyrexia	5 (7.94%) 8	3 (4.76%) 3	0 (0.00%) 0
Immune system disorders	1 (1.59%) 1	3 (4.76%) 3	0 (0.00%) 0
Hypersensitivity	1 (1.59%) 1	3 (4.76%) 3	0 (0.00%) 0
Investigations	1 (1.59%) 1	4 (6.35%) 5	3 (4.76%) 4
Blood creatinine increased	0 (0.00%) 0	2 (3.17%) 2	1 (1.59%) 1
Creatinine increase	0 (0.00%) 0	1 (1.59%) 1	1 (1.59%) 1
Hepatic enzyme increased	1 (1.59%) 1	0 (0.00%) 0	0 (0.00%) 0
Platelet count decreased	0 (0.00%) 0	1 (1.59%) 2	1 (1.59%) 2
Metabolism and nutrition disorders	1 (1.59%) 1	4 (6.35%) 4	0 (0.00%) 0
Hypokalaemia	1 (1.59%) 1	4 (6.35%) 4	0 (0.00%) 0
Musculoskeletal and connective tissue disorders	5 (7.94%) 6	4 (6.35%) 5	0 (0.00%) 0
Back pain	5 (7.94%) 6	4 (6.35%) 5	0 (0.00%) 0
Renal and urinary disorders	0 (0.00%) 0	3 (4.76%) 3	0 (0.00%) 0
Renal impairment	0 (0.00%) 0	3 (4.76%) 3	0 (0.00%) 0
Respiratory, thoracic and mediastinal disorders	1 (1.59%) 1	0 (0.00%) 0	0 (0.00%) 0
Dyspnoea	1 (1.59%) 1	0 (0.00%) 0	0 (0.00%) 0
Skin and subcutaneous tissue disorders	0 (0.00%) 0	2 (3.17%) 2	0 (0.00%) 0
Pruritus	0 (0.00%) 0	1 (1.59%) 1	0 (0.00%) 0
Rash pruritic	0 (0.00%) 0	1 (1.59%) 1	0 (0.00%) 0

N = Number of patients in respective treatment, n = Number of patients in respective categories, e = Number of events, LAmB = liposomal amphotericin B, MF = miltefosine, TEAE = treatment emergent adverse event. Percentages are based on the number of patients allocated to treatment.

## Discussion

This multicountry study is the first clinical trial in PKDL in which a standardized scoring method has been used to measure objectively the extent of the skin area affected. Based on the *post-hoc* analysis, in which an improvement in re-pigmentation of macular lesions of at least 70% was considered clinically meaningful, we observed that 46% and 51% of patients in arms A and B, respectively, had both completely resolved all papular/nodular lesions and had at least 70% of all macular lesions re-pigmented at 12 months. However, this increased to 73% and 80% at 24 months, reflecting the slow recovery of macules to full re-pigmentation, which depends on the regeneration of damaged melanocytes and renewed melanogenesis. Most (93%) patients had either macular or mixed lesions, and healing of these lesions was still ongoing between 12 and 24 months. The scoring method was limited to capturing the areas affected and did not assess partial improvement in skin re-pigmentation (i.e., even a faintly visible PKDL lesion would qualify as a body area affected), therefore, this conservative measurement did not fully capture the improvement of macular PKDL.

Using a qualitative assessment method, similar to that used in previous PKDL trials, overall lesion improvement was assessed by the Investigator considering not only the extension of the area affected but also the improvement in re-pigmentation of the skin. This allowed for a more complete picture of lesion progression, which explains the differences in outcome observed with the two methods. Using the qualitative method, most lesions had either resolved or improved (categories 1 and 2) in 90% of patients in arm A and 85% of patients in arm B at 12 months, whereas at 24 months the proportions were 83% in arm A and 88% in arm B, due to a few cases of relapse.

There is a need to develop and standardize improved methods to quantify lesion progression/improvement in PKDL to allow for a more objective measurement of skin re-pigmentation, especially with the increase in the number of macular cases. Digital technology combined with artificial intelligence as used in other skin diseases should be explored [21], in addition to the development and validation of a parasitological test of cure, ideally antigen or nucleic acid based. Relapses occurred during the 12 to 24-month period: five (5/62, 8.0%) in arm A and one (1/60, 1.7%) in arm B. Varying relapse rates have been reported in PKDL after therapy with LAmB, MF, and combination therapy. Pandey *et al*. [22] found an initial cure rate of 98% (49/50 patients) after treatment and a relapse rate of 26% (12/47 patients) at 12 months with LAmB (total dose of 30 mg/kg) in India, while den Boer et al. [11] found a relapse rate of 2.6% (7/273 patients) at 12 months with LAmB (total dose of 15 mg/kg) in Bangladesh. In Pandey’s study of 12 weeks of MF (100 mg/day), 13% (6/46 patients) relapsed after initial cure at 12 months, and in a study by Ramesh et al., the relapse rate was 15% (11/73 patients) at 18 months with the same MF regimen [22,23]. A later report by Ramesh documented 25% relapses (four patients) at 18 months after initial cure with 12 weeks of MF [16]. In the same study, an MF + LAmB combination (three doses of 5 mg/kg IV LamB over two weeks and 100 mg/kg/day MF for 45 days) resulted in no relapses. All these patients initially responded well to therapy. In studies where qPCR was used to quantify skin parasite load, parasite DNA had all but disappeared at EOT, but was detectable again months to years later when patients relapsed [24]. The main limitation of this study is that it does not allow a direct comparison of the two treatment arms. A comparative study with 12 weeks of miltefosine as a comparator would have been ideal, but infeasible in the current context of low disease incidence thanks to VL elimination efforts in South Asia. Also, given the lack of a standardized measurement for treatment outcome in PKDL, and the diverse definitions of cure used in previous studies, the results of the present study in relation to previous PKDL trials should be interpretated with caution; in this sense, the secondary efficacy endpoint of qualitative assessment by the Investigators used in this trial would be closer to data generated in previous studies. The observed disadvantage of the LamB + MF combination therapy (arm B) is the high frequency of mild MF-related vomiting (albeit only in one patient requiring treatment discontinuation) and the need for women of childbearing potential to have a negative pregnancy test and use contraception for six months. Finally, relapses occurred in both arms, although more frequently in arm A, and increased between 12–24 months. PKDL relapse seems to be unpredictable and happens in patients with initial cure and almost complete clearance of parasites; immunological factors and the initial severity of the lesions and initial high parasite load may play a role [23].

MF monotherapy for 12 weeks has high initial cure rates, but adherence is very poor and exacerbated by gastro-intestinal side effects, and recent reports indicate increasing relapse rate with this regimen [8,9,23]. Moreover, its potential teratogenicity and serious ocular adverse effects after prolonged (12 weeks) treatment are important limitations for this therapy [10,25,26]. MF has been used extensively in the treatment of VL in the ISC, with only rare severe drug-related adverse events, but the treatment duration is short (four weeks). The risk of ocular toxicity, including reports of irreversible blindness, makes this regimen unsuitable for an otherwise healthy individual such as a patient with PKDL; this risk needs to be better characterized and may preclude long-duration use of MF. WHO has recently published special warnings and precautions to minimize ocular adverse events with MF [19]. In the present study, where MF regimen was limited to 3 weeks in combination with LAmB, no ocular toxicity events were observed.

In addition to patient’s benefit, PKDL is treated to reduce the parasite load so that patients are no longer infective to sandflies. Sterile cure is unlikely to be achieved in VL [27], and people with PKDL may have residual parasites detectable after treatment. Eventually, as in VL, it seems inevitable that a certain proportion of people with PKDL will relapse, independent of the treatment given. People with PKDL should be regularly monitored after treatment and instructed to return to hospital if new lesions occur.

No new safety signals were reported, and the safety profile observed in this trial was acceptable. Hypokalaemia was reported in 4.0% of patients but did not necessitate treatment withdrawal. No cases of rhabdomyolysis were observed, as have been described in Bangladesh for patients treated with a total LAmB dose of 30mg/kg [13]. Outpatient treatment with MF was well-tolerated overall, with good rates of treatment adherence.

Both of the regimens studied were suitably safe and efficacious and could serve as alternatives to long-course MF for PKDL in patients from the Indian subcontinent. For sustained elimination of VL as a public health problem, however, there is a need to develop new, safer, short-duration oral therapies for VL and PKDL that can be integrated at primary health centres and deployed on an out-patient basis.

## Supporting information

S1 Text Supplementary material. (DOCX)

S2 Text Consort Checklist. (DOCX)
