# Supplementary material for: A phase II, non-comparative randomised trial of two treatments involving liposomal amphotericin B and miltefosine for post-kala-azar dermal leishmaniasis in India and Bangladesh
Source: PLoS Negl Trop Dis. 2024 Jun 20;18(6):e0012242. doi: 10.1371/journal.pntd.0012242 (PMC11189210; doi:10.1371/journal.pntd.0012242)
Supplement: S1 Text — (DOCX) [file pntd.0012242.s001.docx]

**A phase II, non-comparative randomised trial of two treatments involving liposomal amphotericin B and miltefosine for post kala-azar dermal leishmaniasis in India and Bangladesh**

Supplementary material

**CONTENTS**

[**Table A. Laboratory values at baseline** 2](#_Toc167782080)

[**Table B. Definitive cure at 12 months – mITT population, set of completers** 3](#_Toc167782081)

[**Table C. Proportion of patients with definitive cure at 12 and 24 months for different clinical presentation at baseline (mITT population, worst case scenario)** 3](#_Toc167782082)

[**Table D. Summary of treatment emergent adverse events by system organ class and preferred term – mITT population** 4](#_Toc167782083)

# **Table A. Laboratory values at baseline**

|  | **Statistics** | **Arm A (LAmB) (N = 63)** | **Arm B  (LAmB/MF) (N = 63)** | **Total (N = 126)** |
| --- | --- | --- | --- | --- |
| Haemoglobin (g/dl) | N | 63 | 63 | 126 |
|  | Mean (SD) | 13.1 (1.65) | 12.9 (1.52) | 13.0 (1.58) |
|  | Median | 13.0 | 12.6 | 12.9 |
|  | Min, Max | 9.2, 17.5 | 9.7, 16.9 | 9.2, 17.5 |
| Total WBC Count (/µl) | N | 63 | 63 | 126 |
|  | Mean (SD) | 8,054.0 (2,622.66) | 8,292.1 (2,469.71) | 8,173.0 (2,539.93) |
|  | Median | 7,800.0 | 8,100.0 | 7,900.0 |
|  | Min, Max | 2,800.0, 18,800.0 | 3,600.0, 16,200.0 | 2,800.0, 18,800.0 |
| Platelet count (/µl) | N | 63 | 63 | 126 |
|  | Mean (SD) | 215,206.3 (67,231.70) | 224,269.8 (71,491.53) | 219,738.1 (69,265.77) |
|  | Median | 201,000 | 219,000 | 210,000 |
|  | Min, Max | 87,000, 395,000 | 128,000, 579,000 | 87,000, 579,000 |
| Creatinine (CRE) (mg/dl) | N | 63 | 63 | 126 |
|  | Mean (SD) | 0.9 (0.21) | 0.8 (0.23) | 0.8 (0.22) |
|  | Median | 0.8 | 0.8 | 0.8 |
|  | Min, Max | 0.5, 1.3 | 0.4, 1.4 | 0.4, 1.4 |
| Potassium (mmol/L) | N | 63 | 63 | 126 |
|  | Mean (SD) | 4.1 (0.32) | 4.0 (0.32) | 4.1 (0.32) |
|  | Median | 4.1 | 4.0 | 4.0 |
|  | Min, Max | 3.6, 5.0 | 3.5, 4.9 | 3.5, 5.0 |
| SGOT(AST) (U/L) | N | 63 | 63 | 126 |
|  | Mean (SD) | 30.0 (13.55) | 32.8 (10.48) | 31.4 (12.14) |
|  | Median | 29.2 | 31.0 | 30.3 |
|  | Min, Max | 8.6, 100.2 | 17.0, 81.9 | 8.6, 100.2 |
| SGPT(ALT) (U/L) | N | 63 | 63 | 126 |
|  | Mean (SD) | 27.7 (15.42) | 28.6 (13.70) | 28.2 (14.54) |
|  | Median | 25.1 | 24.6 | 25.1 |
|  | Min, Max | 7.0, 91.3 | 12.3, 84.5 | 7.0, 91.3 |
| Total bilirubin (TBIL) (mg/dl) | N | 63 | 63 | 126 |
|  | Mean (SD) | 0.8 (0.22) | 0.7 (0.23) | 0.7 (0.22) |
|  | Median | 0.8 | 0.8 | 0.8 |
|  | Min, Max | 0.2, 1.7 | 0.1, 1.5 | 0.1, 1.7 |

LAmB = liposomal amphotericin B, MF = miltefosine.

# **Table B. Definitive cure at 12 months – mITT population, set of completers**

| **Outcome** | **Statistics** | **Arm A (LAmB) (N=62)** | **Arm B (LAmB/MF) (N=60)** | **Total (N=122)** | |
| --- | --- | --- | --- | --- | --- |
| Responder | n (%) | 18 (29.03%) | 19 (31.67%) | 37 (30.33%) | |
|  | 95% CI | (17.73%, 40.33%) | (19.90%, 43.44%) | (22.17%, 38.48%) | |
| Non-responder | n (%) | 44 (70.97%) | 41 (68.33%) | 85 (69.67%) | |
|  | 95% CI | (59.67%, 82.27%) | (56.56%, 80.10%) | (61.52%, 77.83%) | |
| **% Improvement for non-responders** | | | | | |
| ≤70% improvement | n (%) | 32 (51.61%) | 25 (41.67%) | 57 (46.72%) |  |
|  | 95% CI | (39.17%, 64.05%) | (29.19%, 54.14%) | (37.87%, 55.57%) |  |
| >70% improvement | n (%) | 11 (17.74%) | 13 (21.67%) | 24 (19.67%) |  |
|  | 95% CI | (8.23%, 27.25%) | (11.24%, 32.09%) | (12.62%, 26.73%) |  |

LAmB = liposomal amphotericin B, MF = miltefosine

Note: Patients withdrawn for AE were considered as non-responders for the 12 months assessment. Patients with missing data at 12 months were not included in the categorization of non-responders.

# **Table C. Proportion of patients with definitive cure at 12 and 24 months for different clinical presentation at baseline (mITT population, worst case scenario)**

| **Clinical form** | **Visit** | **Responders** | | |
| --- | --- | --- | --- | --- |
| Patients with only nodular or papular lesions |  | **Arm A (LAmB)  (N=4) n (%)** | **Arm B (LAmB/MF)  (N=5) n (%)** | **Total (N=9) n (%)** |
|  | 12 Months | 2 (50.00%) | 2 (40.00%) | 4 (44.44%) |
|  | 24 Months | 1 (25.00%) | 3 (60.00%) | 4 (44.44%) |
| Patients with only macular lesions |  | **Arm A (LAmB)   (N=26) n (%)** | **Arm B (LAmB/MF)  (N=25) n (%)** | **Total (N=51) n (%)** |
|  | 12 Months | 11 (42.31%) | 11 (44.00%) | 22 (43.14%) |
|  | 24 Months | 18 (69.23%) | 20 (80.00%) | 38 (74.51%) |
| Patients with mixed lesions |  | **Arm A (LAmB)   (N=32) n (%)** | **Arm B (LAmB/MF)  (N=30) n (%)** | **Total (N=62) n (%)** |
|  | 12 Months | 5 (15.63%) | 6 (20.00%) | 11 (17.74%) |
|  | 24 Months | 15 (46.88%) | 16 (53.33%) | 31 (50.00%) |

LAmB = liposomal amphotericin B, MF = miltefosine

# **Table D. Summary of treatment emergent adverse events by system organ class and preferred term – mITT population**

| **System Organ Class** | **MedDRA (PT) (Version 20.1)** | **Arm A (LAmB) (N=63) n (%) e** | | **Arm B (LAmB/MF) (N=63) n (%) e** | **Total (N=126) n (%) e** |
| --- | --- | --- | --- | --- | --- |
| Gastrointestinal disorders | Abdominal pain upper | | 0 (0.00%) 0 | 1 (1.59%) 1 | 1 (0.79%) 1 |
|  | Diarrhoea | 0 (0.00%) 0 | | 1 (1.59%) 1 | 1 (0.79%) 1 |
|  | Gastritis | 0 (0.00%) 0 | | 1 (1.59%) 1 | 1 (0.79%) 1 |
|  | Haematemesis | 0 (0.00%) 0 | | 1 (1.59%) 1 | 1 (0.79%) 1 |
|  | Nausea | 1 (1.59%) 1 | | 6 (9.52%) 7 | 7 (5.56%) 8 |
|  | Toothache | 0 (0.00%) 0 | | 1 (1.59%) 1 | 1 (0.79%) 1 |
|  | Vomiting | 1 (1.59%) 1 | | 38 (60.32%) 130 | 39 (30.95%) 131 |
| General disorders and administration site conditions | Asthenia | 0 (0.00%) 0 | | 1 (1.59%) 1 | 1 (0.79%) 1 |
|  | Chills | 1 (1.59%) 1 | | 1 (1.59%) 1 | 2 (1.59%) 2 |
|  | Death | 0 (0.00%) 0 | | 1 (1.59%) 1 | 1 (0.79%) 1 |
|  | Pyrexia | 5 (7.94%) 8 | | 3 (4.76%) 3 | 8 (6.35%) 11 |
| Immune system disorders | Hypersensitivity | 1 (1.59%) 1 | | 3 (4.76%) 3 | 4 (3.17%) 4 |
| Infections and infestations | Oral herpes | 0 (0.00%) 0 | | 1 (1.59%) 1 | 1 (0.79%) 1 |
| Investigations | Blood creatinine increased | 1 (1.59%) 1 | | 2 (3.17%) 2 | 3 (2.38%) 3 |
|  | Hepatic enzyme increased | 1 (1.59%) 1 | | 0 (0.00%) 0 | 1 (0.79%) 1 |
|  | Platelet count decreased | 0 (0.00%) 0 | | 1 (1.59%) 2 | 1 (0.79%) 2 |
|  | Creatinine increase | 0 (0.00%) 0x | | 1 (1.59%) 1 | 1 (0.79%) 1 |
| Metabolism and nutrition disorders | Decreased appetite | 1 (1.59%) 1 | | 1 (1.59%) 1 | 2 (1.59%) 2 |
|  | Hypokalaemia | 1 (1.59%) 1 | | 4 (6.35%) 4 | 5 (3.97%) 5 |
| Musculoskeletal and connective tissue disorders | Back pain | 5 (7.94%) 6 | | 4 (6.35%) 5 | 9 (7.14%) 11 |
| Renal and urinary disorders | Renal impairment | 0 (0.00%) 0 | | 3 (4.76%) 3 | 3 (2.38%) 3 |
| Respiratory, thoracic and mediastinal disorders | Dyspnoea | 1 (1.59%) 1 | | 0 (0.00%) 0 | 1 (0.79%) 1 |
| Skin and subcutaneous tissue disorders | Pruritus | 1 (1.59%) 1 | | 1 (1.59%) 1 | 2 (1.59%) 2 |
|  | Rash pruritic | 0 (0.00%) 0 | | 1 (1.59%) 1 | 1 (0.79%) 1 |
| Vascular disorders | Hypertension | 1 (1.59%) 1 | | 0 (0.00%) 0 | 1 (0.79%) 1 |
| **Total** | | **18 (28.57%) 25** | | **49 (77.78%) 172** | **67 (53.17%) 197** |

LAmB = liposomal amphotericin B, MF = miltefosine. N = Number of patients in respective treatment. n = Number of patients in respective categories; e = Number of events. Percentages are based on the number of patients allocated to treatment.
